# Telerehabilitation for Fall Prevention in Saudi Arabia: Readiness and Predictors Among Physical Therapists

**DOI:** 10.3390/jcm14217838

**Published:** 2025-11-04

**Authors:** Saad M. Bindawas, Hussam M. Alsaleh, Vishal Vennu

**Affiliations:** Department of Rehabilitation Sciences, College of Applied Medical Sciences, King Saud University, Riyadh 10219, Saudi Arabia; 443106482@student.ksu.edu.sa (H.M.A.); vvennu@ksu.edu.sa (V.V.)

**Keywords:** physical therapy, digital health interventions, remote rehabilitation, geriatric injury prevention, healthcare workforce readiness, Middle Eastern healthcare

## Abstract

**Background:** Telerehabilitation can extend fall prevention to underserved groups. Clinician uptake remains limited. We evaluated factors associated with physical therapists’ belief in and readiness to implement the Otago Exercise Program (OEP) via telerehabilitation in Saudi Arabia. **Methods:** We conducted a national cross-sectional survey of physical therapists (*n* = 120). Exposures included fall-prevention training, education outside versus inside Saudi Arabia, and familiarity with telerehabilitation. Outcomes were belief in telerehabilitation efficacy for the OEP (5-point Likert) and high readiness, defined as familiarity plus knowledge ≥4 of 5 and belief ≥4 of 5. We utilized ordinal logistic regression for assessing belief and binary logistic regression for assessing readiness. We conducted two-sided tests with alpha equal to 0.05 and prespecified covariates. Sensitivity analyses using penalized likelihood methods confirmed the robustness of the results. **Results:** The OEP prescription did not differ with telerehabilitation familiarity (χ^2^ = 0.28; *p* = 0.597). In multivariable models using complete cases (*n* = 117; 21 readiness events), fall-prevention training was associated with higher belief (odds ratio [OR] = 3.997; 95% CI = 1.568–10.184; *p* = 0.004) and higher readiness (OR = 4.520; 95% CI = 1.546–13.210; *p* = 0.006). Education outside Saudi Arabia was associated with higher readiness (OR = 5.170; 95% CI = 1.435–18.636; *p* = 0.012). Results were directionally consistent in Firth and pilot-exclusion analyses. **Conclusions:** Training and international educational exposure were associated with stronger beliefs and greater readiness to implement the OEP via telerehabilitation. Basic familiarity alone was not associated with the OEP use. Programs should prioritize competency-based training and curricular updates to support implementation.

## 1. Introduction

Falls are a leading cause of injury, disability, and loss of independence in older adults, with substantial effects on quality of life and healthcare use in rapidly aging regions, including the Middle East [1,2,3]. The Otago Exercise Program (OEP) is a progressive strength and balance program that reduces falls and fall-related injuries and can be utilized for remote support, extending access where in-person services are constrained [4,5,6,7]. Telerehabilitation is a practical modality to scale evidence-based exercise for community-dwelling older adults [8].

Successful telerehabilitation requires competent clinicians and supportive organizational and system factors. The American Physical Therapy Association (APTA) Telerehabilitation Clinical Practice Guideline (2024) emphasizes competency-based preparation, workflow and documentation standards, monitoring and safety, and outcome evaluation as prerequisites for safe and effective delivery in physical therapist practice [9]. Complementing professional guidance, the World Health Organization (WHO) Package of Interventions for Rehabilitation (2023) specifies service-level requirements (workforce skills and assistive products/equipment) for prioritized interventions, and the WHO Global Strategy on Digital Health 2020–2025, together with Rehabilitation 2030, situates digital rehabilitation within broader health system strengthening in governance, interoperability, financing, and human resources [10,11,12]. Collectively, these frameworks suggest that clinician training and educational exposure, rather than awareness alone, are likely levers for adoption.

To examine the determinants of clinician adoption, we applied the Unified Theory of Acceptance and Use of Technology (UTAUT). The UTAUT posits that adoption reflects Performance Expectancy (anticipated benefit), Effort Expectancy (perceived ease), Social Influence (norms), and Facilitating Conditions (training and infrastructure). In our dataset, the belief that telerehabilitation can effectively deliver the OEP operationalizes Performance Expectancy, and fall-prevention training plus educational background (degree earned outside vs. inside Saudi Arabia) approximate Facilitating Conditions. Familiarity with telerehabilitation is treated as a prior-exposure indicator rather than an Effort Expectancy proxy because ease-of-use items were not collected; Effort Expectancy and Social Influence are acknowledged gaps to be addressed prospectively.

A prior national work in Saudi Arabia established excellent internal consistency and test–retest reliability for the underlying survey and described therapists’ OEP knowledge and attitudes [5]. However, the theory-aligned predictors of telerehabilitation-based OEP implementation have not been examined. It remains unclear whether familiarity translates into use or whether training and educational exposure more strongly shape belief and readiness to implement.

Guided by the UTAUT and consistent with the APTA and WHO implementation guidance, this study had three specific aims. First, we tested whether familiarity with telerehabilitation was associated with prior OEP prescriptions, examining whether exposure translates into use. Second, we identified predictors of belief in the efficacy of telerehabilitation for delivering the OEP, operationalizing the UTAUT’s Performance Expectancy construct. Third, we evaluated predictors of high readiness to implement remote fall prevention, defined as the conjunction of familiarity, adequate knowledge, and strong belief, serving as a behavioral-intention proxy. We hypothesized that specialized training and international educational exposure would independently predict both belief and readiness, while familiarity alone would not predict historical use.

## 2. Materials and Methods

### 2.1. Study Design and Data Source

We conducted a national, cross-sectional online survey of licensed physical therapists (PTs) practicing in Saudi Arabia between November 2023 and April 2024. The survey characterized the use of telerehabilitation to deliver the Otago Exercise Program (OEP) to older adults and identified the determinants of belief (perceived efficacy) and readiness to implement the OEP via telerehabilitation. Reporting followed the STROBE guidance for observational studies (checklist available on request). Sampling, instrument development, and model specifications were prespecified and are described below.

### 2.2. Participants and Recruitment

Eligible participants were licensed PTs practicing in Saudi Arabia. PT students, interns, and nonlicensed personnel were excluded. Recruitment used convenience channels, including professional associations and groups, departmental mailing lists, and social media. Denominator counts for outreach channels were unavailable; a response rate could not be calculated. Generalizability is therefore limited, and selection bias is possible, as noted in the Discussion. Demographic and professional characteristics are shown in Table 1.

### 2.3. Instrument Development and Validation

The survey was adapted from a prior national work in Saudi Arabia that reported strong content and face validity and high reliability for the OEP and telerehabilitation items (knowledge Cronbach α ≈ 0.98; attitudes α ≈ 0.97; two-week test–retest ICC ≈ 0.92–0.93) [5]. Before rollout, a four-member expert panel (geriatrics, digital health, rehabilitation) conducted a content review, and clinicians with ≥7 years of experience provided additional face validity feedback. A pilot study (*n* = 30) confirmed reliability and usability. Instrument content and administration were identical in the pilot and main phases; data were pooled for analysis with a prespecified pilot-exclusion sensitivity (2.8). Scale development and reliability followed health-measurement best practices [13,14].

The instrument included the following: Section A, demographic/professional characteristics (14 items); Section B, OEP knowledge (15 items); and Section C, attitudes toward the OEP and telerehabilitation (13 Likert-type items; 1 = strongly disagree to 5 = strongly agree, plus “not applicable”). Item wording is provided in the prior publication and the Supplement [5].

### 2.4. Measures and UTAUT Operationalization

Determinants were structured using the UTAUT [15].

#### 2.4.1. Outcomes

- The OEP prescription (use behavior): ever prescribed the OEP to older adults (yes/no).
- Belief that telerehabilitation can effectively deliver the OEP (Performance Expectancy): single 5-point Likert item (higher = stronger belief), analyzed with ordinal logistic regression.
- Readiness to implement the OEP via telerehabilitation (behavioral-intention proxy): binary composite (definition in 2.5).

#### 2.4.2. Predictors and Covariates

- Telerehabilitation familiarity (exposure indicator): familiar vs. not familiar (binary).
- Fall-prevention training/certification (Facilitating Conditions): yes/no.
- Educational background (Facilitating Conditions): highest degree earned outside vs. inside Saudi Arabia.
- Practice context: sector/setting and region of practice.
- Demographics: age group, sex, and years in practice (categorical).

#### 2.4.3. UTAUT Mapping

In this dataset, belief operationalized Performance Expectancy, and training and educational background approximated Facilitating Conditions. Familiarity was treated as prior exposure (not an Effort Expectancy proxy) because ease-of-use items were not collected. Effort Expectancy and Social Influence were not measured and are identified as gaps for future work. Construct-to-variable mapping and coding are summarized in Table 1 [15].

#### 2.4.4. UTAUT Operationalization

We used the UTAUT to structure determinants of telerehabilitation adoption. Performance Expectancy was represented by the 5-point belief item (“telerehabilitation can effectively deliver OEP”). Facilitating Conditions were approximated by fall-prevention training/certification, educational background (outside vs. inside Saudi Arabia), and practice context. Familiarity was treated as prior exposure. Effort Expectancy and Social Influence were not directly measured and are acknowledged as limitations (Table 1).

**Table 1:** UTAUT mapping.

UTAUT Construct	Definition (Clinician Adoption)	Measured Variable(s)	Coding/ Cut-Points	Model Role	Gap and Next-Step Measure
Performance Expectancy	Belief technology improves outcomes/efficiency	“Telerehabilitation can effectively deliver OEP”	Likert 1–5 (higher = stronger)	Outcome (belief); descriptive in readiness framing	Add outcome-expectancy items specific to tele-exercise
Effort Expectancy	Perceived ease of use	Not directly measured	—	—	Add usability/ease-of-use items (UTAUT-adapted/SUS-style)
Social Influence	Peer/leader endorsement	Not directly measured	—	—	Add supervisor/peer norms and patient preference items
Facilitating Conditions	Training/infrastructure/support enabling use	Fall-prevention training (Y/N); education outside vs. inside the KSA; sector/setting	Binary/categorical	Predictors (belief, readiness)	Add items on platform access, IT/helpdesk, reimbursement clarity
Behavioral Intention	Intention to use	Readiness composite (main: familiarity and knowledge ≥4 and belief ≥4)	Binary (ready vs. not)	Outcome (not used as a predictor)	Consider a separate intention scale with behavioral anchors
Use Behavior	Actual adoption	Prior OEP prescription (Y/N)	Binary	Outcome	Add frequency/intensity of OEP delivery; tele-only vs. hybrid
Exposure indicator (not UTAUT)	Prior exposure/experience	Familiarity with telerehabilitation	Binary	Covariate: predictor of use	Keep as exposure; avoid misclassifying as Effort Expectancy

Abbreviations: KSA, Kingdom of Saudi Arabia; OEP, Otago Exercise Program; UTAUT, Unified Theory of Acceptance and Use of Technology; IT, information technology. Rationale: familiarity is prior exposure and not ease of use.

### 2.5. Readiness Definition and Rationale

“High telerehabilitation readiness” required the fulfillment of the following three criteria: (i) familiarity with telerehabilitation, (ii) knowledge ≥ 4/5, and (iii) belief ≥ 4/5. This conjunctive threshold reflects implementation practice, in which exposure and favorable appraisal are prerequisites for safe clinical deployment. To mitigate circularity, the primary readiness model excluded knowledge and belief as predictors (anti-tautology rule). As secondary, exploratory specifications, we evaluated models that included knowledge and belief and a “two-of-three” readiness threshold; qualitative inferences were unchanged.

### 2.6. Data Management and Missing Data

Data were screened for duplicates and implausible entries using time-stamp, IP, and email heuristics; none were identified. Item-level missingness was low. Analyses used complete-case data for each model. Model-specific sample sizes are reported in table footnotes. The prespecified category structure was retained. Instability due to sparse cells was addressed through penalized likelihood in sensitivity analyses and with cautious interpretation.

### 2.7. Statistical Analysis

**Figure 1:**
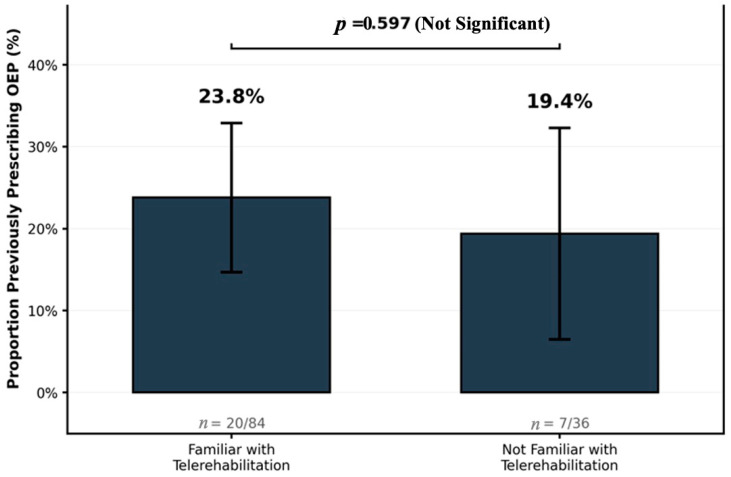
Percentage of PTs who have prescribed the OEP, stratified by self-reported familiarity with telerehabilitation. Proportions are shown with counts. Difference not significant (χ^2^(1) = 0.28; *p* = 0.597). Note: Data correspond to Table 3.

All tests were two-sided with α = 0.05. Descriptive statistics summarized participant characteristics (Table 2). The OEP use by familiarity was compared using Pearson χ^2^ (degrees of freedom and exact *p* reported; Table 2 and Figure 1).

#### 2.7.1. Modeling Strategy

- Belief (5-level Likert) was modeled with ordinal logistic regression (proportional odds) using prespecified covariates [16].
- The OEP prescription and readiness (binary) were modeled with logistic regression using prespecified covariates. The primary readiness model included training and education outside/inside Saudi Arabia as focal predictors and excluded knowledge and belief to avoid tautology.

#### 2.7.2. Diagnostics and Fit

- Multicollinearity was assessed using variance inflation factors (VIFs). In an extended readiness model that included belief, VIFs ranged from 1.13 to 1.72 (Supplementary Table S2).
- Goodness of fit was evaluated using the Akaike Information Criterion (AIC) and the Hosmer–Lemeshow test for logistic models; ordinal models reported the AIC and pseudo-R^2^ [17].
- The proportional odds assumption was tested for ordinal models (Brant-type test). If violated, a partial proportional odds model was prespecified [16,18].
- Where helpful, bootstrap 95% CIs (1000 resamples) are reported in the Supplement to illustrate the estimated precision in small-event settings [19].

#### 2.7.3. Software

We conducted analyses in Excel, SPSS version 27, and R version 4.4.2. Penalized likelihood logistic regression used Firth’s method [20,21].

### 2.8. Sensitivity Analyses (Small-Event Robustness)

Given that 21 participants met the readiness criterion, we prespecified two robustness checks: (1) Firth’s penalized logistic regression to reduce small-sample bias and address potential separation [20,21] and (2) pilot-exclusion analysis to re-estimate the readiness model after removing pilot respondents (n = 30; main-phase sample n = 87; readiness events n = 16). Both analyses used the same covariates as the primary readiness model and are summarized in Supplementary Material S1, Table S1. As reported in the Results and Supplement, training and education outside Saudi Arabia remained statistically significant with directionally consistent magnitudes (for example, Firth: training OR = 3.74; university outside OR = 4.15; no-pilot: training OR = 4.85; university outside OR = 5.35), supporting robustness to small-event bias and pilot inclusion.

### 2.9. Ethical Considerations

The study complied with the Declaration of Helsinki and local regulations. Electronic informed consent was obtained from all participants before they were granted access to the survey. The Institutional Review Board of King Saud University approved the study protocol (KSU–HE–23–387; approval date: 11 April 2023). Only de-identified data were analyzed; anonymity was maintained throughout.

## 3. Results

### 3.1. Participant Characteristics

Among 120 licensed PTs surveyed nationwide, specialized fall-prevention training and international educational exposure emerged as independent predictors of readiness to implement remote fall-prevention programs. In contrast, familiarity with telerehabilitation alone did not predict prior use of the OEP. These key findings and supporting analyses are detailed below.

Participants were 57.5% male (*n* = 69). Most were aged 25–34 years (60.0%, *n* = 72), followed by 35–44 years (25.0%, *n* = 30). By region, the Central area had the highest representation (46.7%, *n* = 56), followed by Northern (19.2%, *n* = 23). Highest qualifications were bachelor’s (49.2%), master’s (35.8%), PhD (11.7%), clinical doctorate (2.5%), and postgraduate diploma (0.8%). Overall, 28.3% of the participants (*n* = 34) obtained their highest degree outside Saudi Arabia. Clinical experience was ≤5 years in 44.1%, 6–10 years in 25.0%, and ≥11 years in 30.8% of the participants. In total, 35.8% of the participants (*n* = 43) reported fall-prevention training, 70.0% (*n* = 84) reported familiarity with telerehabilitation, and 22.5% (*n* = 27) had been prescribed the OEP (Table 2).

**Table 2:** Participant characteristics (N = 120).

Characteristic	Category	*n*	%
Gender	Male	69	57.5%
	Female	51	42.5%
Age	<25	8	6.7%
	25–34	72	60.0%
	35–44	30	25.0%
	45–54	9	7.5%
	55–64	1	0.8%
Region	Central	56	46.7%
	Northern	23	19.2%
	Southern	21	17.5%
	Western	17	14.2%
	Eastern	3	2.5%
Highest Qualification	Bachelor degree	59	49.2%
	Master degree	43	35.8%
	PhD	14	11.7%
	Clinical doctorate	3	2.5%
	Postgraduate diploma	1	0.8%
University Location	University in Saudi Arabia	86	71.7%
	University outside Saudi Arabia	34	28.3%
Years of Clinical Experience	<2 years	13	10.8%
	2–5 years	40	33.3%
	6–10 years	30	25.0%
	11–15 years	12	10.0%
	16–20 years	12	10.0%
	>20 years	13	10.8%
Fall Prevention Training	No	77	64.2%
	Yes	43	35.8%
Familiar with Telerehab	No	36	30.0%
	Yes	84	70.0%
Previously Prescribed OEP	No	93	77.5%
	Yes	27	22.5%

Abbreviations: OEP, Otago Exercise Program; PhD, Doctor of Philosophy.

### 3.2. OEP Use by Telerehabilitation Familiarity

In the bivariate analysis, the OEP prescriptions did not differ by familiarity with telerehabilitation (Table 3; χ^2^(1) = 0.28; *p* = 0.597). Among the participants familiar with telerehabilitation, 23.8% (20/84) had prescribed the OEP when compared with 19.4% (7/36) of participants unfamiliar with telerehabilitation. Figure 1 shows this non-significant difference.

### 3.3. Predictors of Belief in Telerehabilitation Effectiveness (Ordinal Model)

Multivariable ordinal logistic regression (*n* = 117 complete cases) examined predictors of belief that telerehabilitation can effectively deliver the OEP (Performance Expectancy). Fall-prevention training was associated with higher belief (odds ratio [OR] = 3.997; 95% CI = 1.568–10.184; *p* = 0.004). Familiarity with telerehabilitation was not associated with belief (OR = 0.921; 95% CI = 0.410–2.068; *p* = 0.841). Clinical experience (6–10 years; >10 years vs. <5 years) was not significant (Table 4). These results indicate that formal training, rather than simple exposure, aligns with higher performance expectations for telerehabilitation in the OEP.

### 3.4. Predictors of High Telerehabilitation Readiness (Binary Model)

Readiness was defined a priori as familiarity with telerehabilitation, knowledge ≥ 4/5, and belief ≥ 4/5. In the primary multivariable logistic model (*n* = 117; 21 “ready” events), fall-prevention training and education outside Saudi Arabia were independently associated with higher odds of readiness: training (OR = 4.520; 95% CI = 1.546–13.210; *p *= 0.006) and university outside Saudi Arabia (OR = 5.170; 95% CI = 1.435–18.636; *p *= 0.012). Compared with high knowledge, moderate knowledge was associated with higher readiness (OR = 10.309; 95% CI = 1.276–83.336; *p *= 0.029), whereas low knowledge was not significant (OR = 5.829; *p *= 0.123). Belief categories trended in the expected direction (e.g., high vs. low belief OR = 8.680; *p *= 0.064), but were not significant with α = 0.05 in this specification; belief and knowledge contribute to the readiness definition and were handled to avoid tautology. Neither clinical experience nor region was associated with readiness (Table 5). 

### 3.5. Model Diagnostics, Multicollinearity, and Assumption Checks

Variance inflation factors (VIFs) were computed in an extended readiness model that included belief. VIFs ranged from 1.13 to 1.72 (highest for university outside vs. local = 1.72), indicating low multicollinearity (Supplementary Table S2). Proportional odds testing supported the use of the ordinal specification for belief (test details available on request). Goodness of fit was assessed using the Akaike Information Criterion (AIC) and Hosmer–Lemeshow test for logistic models; ordinal models reported the AIC and pseudo-R^2^ [17]. Diagnostics were consistent with an acceptable fit given the small-event constraints. In accordance with STROBE, exact *p*-values and 95% CIs are reported in Table 3, Table 4 and Table 5, and model-specific sample sizes are stated.

### 3.6. Sensitivity Analyses for Small-Event Robustness

Two prespecified sensitivity analyses addressed the limited number of readiness events (*n* = 21). First, Firth’s penalized logistic regression yielded attenuated but statistically significant estimates for training (OR = 3.74; *p *= 0.010) and education outside Saudi Arabia (OR = 4.15; *p *= 0.018). Second, pilot-exclusion analyses (main-phase sample only, *n* = 87; 16 ready events) produced consistent associations: training (OR = 4.85; *p *= 0.025) and university outside (OR = 5.35; *p *= 0.037). These checks suggest that the primary findings are robust considering a small-sample bias and not driven by the pilot subgroup (Supplementary Material S1, Table S1).

### 3.7. Ancillary and Figure-Based Findings

**Figure 2:**
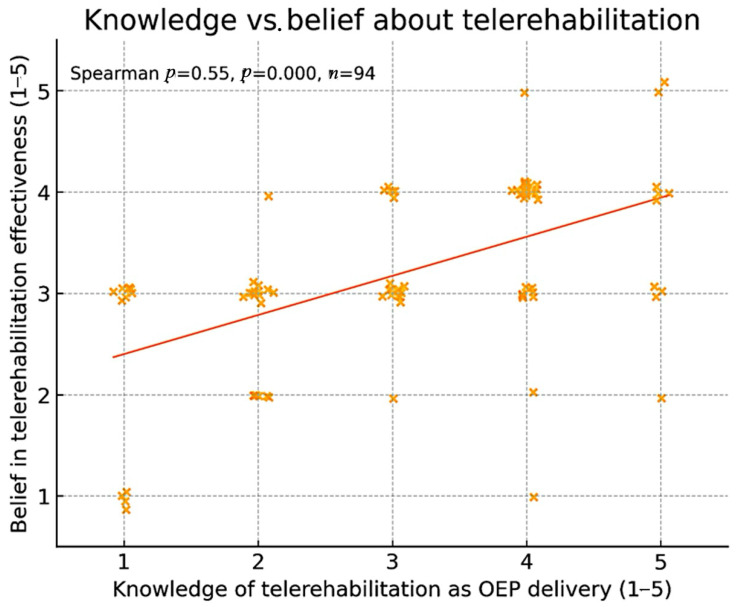
Scatterplot of Likert scores (1–5) for self-rated knowledge versus belief that telerehabilitation can effectively deliver the OEP. Spearman ρ = 0.26; *p *= 0.004. Higher values indicate greater knowledge or belief. Abbreviations: OEP, Otago Exercise Program; ρ, Spearman correlation coefficient.

Exploratory correlation analysis showed a weak but statistically significant positive association between self-rated knowledge of telerehabilitation and belief in its effectiveness (Spearman ρ = 0.26; *p *= 0.004), as depicted in Figure 2. 

**Figure 3:**
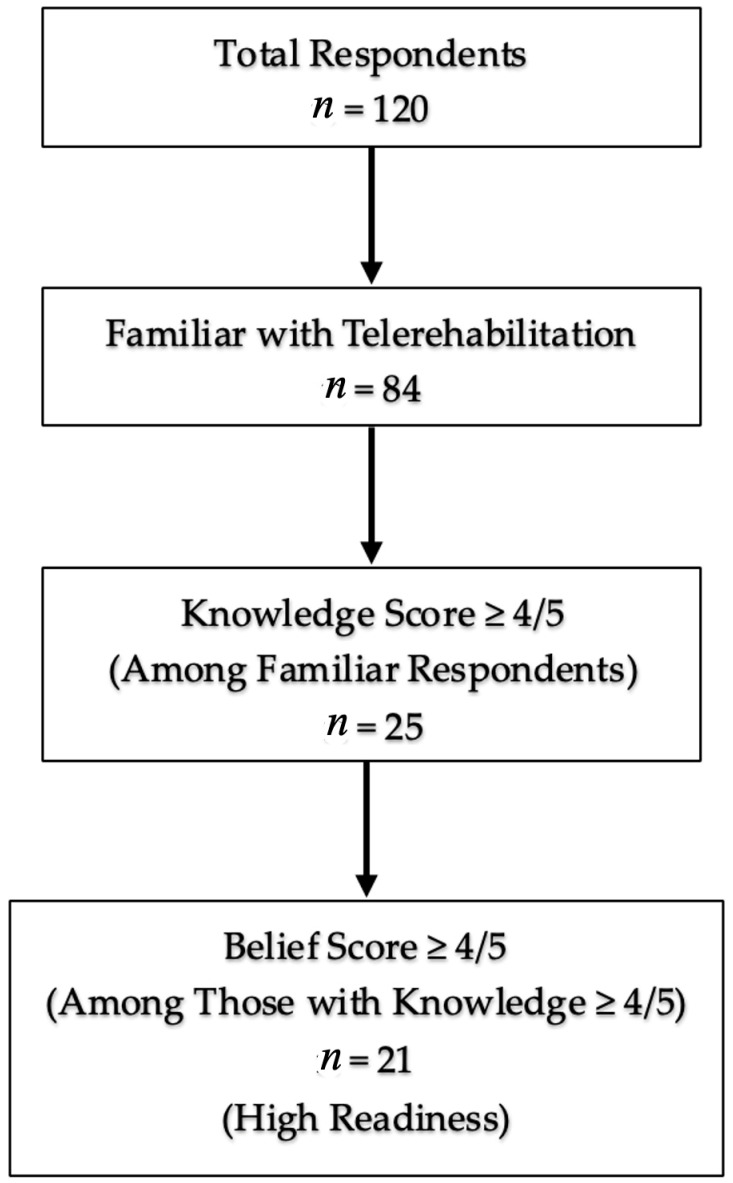
Flow diagram from the total sample to the subgroup classified as “high readiness,” defined a priori as meeting all three criteria: familiarity with telerehabilitation, knowledge ≥ 4/5, and belief ≥ 4/5.

**Figure 4:**
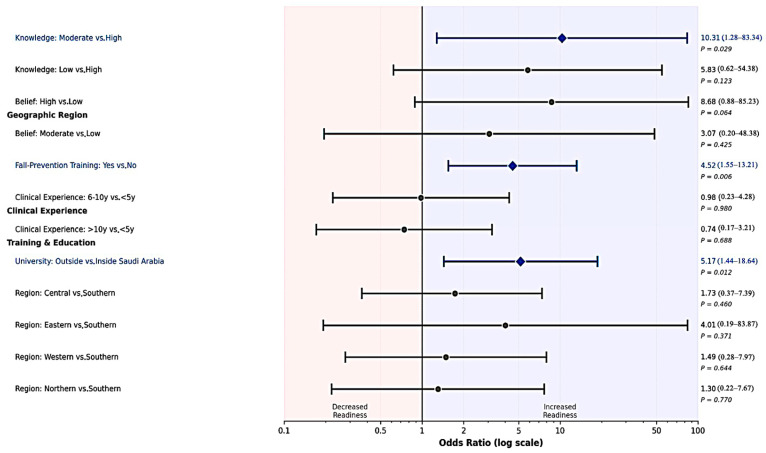
Forest plot showing adjusted odds ratios and 95 percent confidence intervals for predictors of high telerehabilitation readiness. Results from multivariable logistic regression are provided (*n* equals 117; 21 readiness events).

Figure 3 shows the derivation of the high-readiness group (*n* = 21) from the total sample (*n* = 120) based on the predefined criteria (familiarity, knowledge, belief). Figure 4 provides a visual summary of these multivariable associations, highlighting that fall-prevention training and education outside Saudi Arabia emerged as the strongest independent predictors of readiness.

### 3.8. Missing Data and Analytic Samples

Complete-case analyses for regression models yielded *n* = 117, indicating minimal item-level missingness from the full sample of 120. Model-specific sample sizes are reported in table footnotes to facilitate reproducibility and appraisal of precision (Table 3, Table 4 and Table 5).

## 4. Discussion

This study challenges the assumption that digital familiarity alone drives clinical adoption of telerehabilitation. Among licensed Saudi physical therapists, specialized fall-prevention training and international educational exposure, not simple awareness, predicted readiness to implement evidence-based remote fall prevention. These findings have direct implications for health system investments: awareness campaigns are unlikely to shift practice without competency-based training and curricular reform targeted to telerehabilitation skills.

In brief, (1) telerehabilitation familiarity was not associated with the OEP prescription in the bivariate analysis; (2) fall-prevention training was independently associated with stronger belief that the OEP can be delivered effectively via telerehabilitation; and (3) training and earning a highest degree outside Saudi Arabia were independently associated with higher odds of high readiness in multivariable models, with robust results obtained from Firth penalization and pilot-exclusion sensitivity analyses.

Taken together, the results align with the implementation theory: competency and enabling conditions drive adoption more than exposure alone. They extend the Saudi literature on the OEP knowledge and attitudes by identifying theory-aligned determinants of intention to implement the OEP through digital modalities [5].

### 4.1. Principal Findings in the Context of Existing Evidence

The null association between familiarity with telerehabilitation and OEP prescription (χ^2^ = 0.28; *p* = 0.597) challenges the assumption that awareness or exposure translates into use. Prior evidence across rehabilitation and broader health IT domains shows that passive familiarity is often insufficient to change behavior without structured training, workflow integration, and supportive organizational conditions. Clinical practice guidance from the APTA emphasizes competency-based preparation (technology setup, remote examination skills, safety monitoring, documentation, and outcome evaluation) as a prerequisite for safe and effective telerehabilitation, which includes the elements that extend beyond simple exposure [22]. Our results are consistent with this guidance and its implication that readiness is built and not assumed [9].

The association between fall-prevention training and increased belief in the effectiveness of telerehabilitation for the OEP is theoretically and empirically sound. The efficacy of OEP in reducing falls among community-dwelling older adults is well established in randomized trials and meta-analyses [23,24,25,26]. Clinicians who have completed structured training are more likely to understand intervention components, dosing, safety thresholds, and outcome tracking. These competencies likely enhance confidence that the OEP can be delivered remotely without compromising outcomes [27]. Digital adaptations of the OEP have also demonstrated feasibility and safety for remote use among older adults [5,28].

At the system level, global policy frameworks integrate telerehabilitation within health system strengthening and highlight the importance of workforce capacity and structural supports. The WHO Package of Interventions for Rehabilitation specifies service-level requirements, including workforce skills and assistive products, for prioritized interventions [10]. The WHO Global Strategy on Digital Health 2020–2025 and Rehabilitation 2030 situate digital care within governance, financing, interoperability, and human resources reform [11,12]. Our finding that training and educational context—rather than familiarity—predict readiness aligns with these frameworks’ focus on capacity building and conducive conditions [29].

International educational exposure, independent of training, knowledge, and experience, as a predictor of readiness, suggests that curricula, clinical placements, and academic environments encountered abroad may foster durable digital-health competencies and an implementation mindset. Post-pandemic curricula in North America, Europe, and Australasia increasingly embed telehealth assessment, patient monitoring, and documentation [30]. This observation is consistent with diffusion-of-innovation theories and implementation frameworks, such as CFIR, which emphasize individuals’ knowledge and beliefs, as well as the roles of inner setting (culture, learning climate) and outer setting (policy, payment) in adoption [29].

### 4.2. Interpretation Through the UTAUT and CFIR Lenses

We prespecified the UTAUT as the organizing framework. In this dataset, the belief that telerehabilitation can effectively deliver the OEP operationalizes Performance Expectancy, and fall-prevention training and educational background approximate Facilitating Conditions [31]. Effort Expectancy and Social Influence were not directly measured and are acknowledged gaps.

Within this framework, the data suggest that Facilitating Conditions may be proximal drivers of adoption: those with formal fall-prevention training and those educated outside Saudi Arabia—contexts in which digital-health competencies may be integrated into curricula—were more likely to report readiness. This pattern is consistent with the UTAUT’s premise that supportive conditions (training, resources, organizational support) enable users to translate expectations into behavior and with the APTA guideline’s emphasis on competency-based preparation [15].

CFIR complements this view by emphasizing multilevel determinants. Even when individuals hold favorable beliefs, adoption may be hindered if inner-setting features (leadership engagement, learning climate, IT support, workflow) are not aligned with outer-setting factors (reimbursement for virtual visits, licensing, data privacy regulations) [32]. This may explain why training predicted belief and readiness, whereas familiarity did not correlate with prescription behavior in the bivariate analysis. In short, exposure alone does not produce practice change; competency development, along with organizational and policy support, is required [29].

### 4.3. Competence Versus Exposure: Why Familiarity Is Not Enough

The operational distinction between “familiarity” (prior exposure) and “training” (competence building) clarifies the mechanisms underlying our results. Familiarity may reduce anxiety or perceived effort—the elements akin to Effort Expectancy—but it does not confer protocol knowledge (exercise progression, contraindications), remote assessment skills (safety checks, camera positioning, validated performance measures), or proficiency with documentation and data security required for compliant telepractice. Structured training can provide these elements, close the knowledge-to-practice gap, and strengthen confidence in the fact that remote OEP delivery is safe and effective, hence increasing Performance Expectancy among trained clinicians. This interpretation aligns with telerehabilitation evidence syntheses, showing clinical equivalence or noninferiority to in-person delivery when programs are implemented with adequate preparation and support [33].

Digital delivery of fall-prevention exercise is feasible and effective when adherence and progression are supported. StandingTall, a home-based eHealth balance exercise program, reduced falls among community-dwelling older adults in a large randomized trial, reinforcing the plausibility of remote strength and balance interventions for fall risk reduction [34]. Such trials elevate, rather than diminish, the importance of clinician training to translate evidence into routine telepractice.

### 4.4. Educational Context and Readiness: Potential Mechanisms

The independent association between an international highest degree and readiness may reflect several mechanisms: curricular exposure to telehealth competencies; clinical placements involving hybrid or remote care; academic cultures emphasizing evidence-based practice, quality improvement, and interprofessional teamwork; and organizational matching, in which internationally educated therapists work in settings with stronger IT infrastructure and leadership support for digital care. Although our survey was not designed to test these pathways, the association remained significant after Firth penalization and after excluding pilot data, indicating a robust signal that merits qualitative follow-up and multilevel modeling [9].

### 4.5. Methodological Considerations

A small number of “high readiness” events (*n* = 21) limit precision and raise the risk of overfitting. We mitigated these concerns by prespecifying an exploratory interpretation, applying Firth’s penalized logistic regression to reduce small-sample bias and address quasi-separation (yielding attenuated yet significant odds ratios for training and international education), and conducting a pilot-exclusion sensitivity analysis with consistent results [34]. VIFs were low (≈1.13–1.72) in the specification most prone to overlap (including belief), suggesting that multicollinearity is not a driver. These diagnostics support the reliability of the direction of associations, while wide confidence intervals underscore the need for replication to refine effect sizes [29].

Our readiness definition—requiring familiarity > 4/5, knowledge ≥ 4/5, and belief ≥4/5—reflects a conservative implementation stance in which exposure and favorable appraisal are prerequisites for safe deployment. As knowledge and belief contribute to the outcome, we excluded them from the primary readiness model to avoid tautology; exploratory specifications that included them were labeled as hypothesis-generating. Future work should validate a readiness scale independent of these components (for example, behavioral-intention instruments or objective implementation behavior such as counts of OEP tele-prescriptions over time). The future UTAUT-based studies in this population should incorporate validated scales for the remaining original constructs, specifically Effort Expectancy and Social Influence, to provide a more complete model of technology adoption.

Convenience sampling and the absence of a response-rate denominator limit generalizability; respondents may be more academically engaged or more digitally inclined than the broader PT population. The cross-sectional design precludes causal inference. Self-reporting may introduce social desirability or recall bias. These limitations are common in early implementation studies [35] and should be addressed in future research using probability sampling, longitudinal follow-up, and objective behavioral outcomes.

### 4.6. Implications for Practice, Education, and Policy

Awareness campaigns are insufficient to increase the implementation of OEP via telerehabilitation. Clinicians and service leaders should prioritize competency-based training aligned with the APTA telerehabilitation guidelines, covering remote assessment workflows, exercise progression and safety, documentation standards, emergency procedures, and outcome evaluation [9]. Embedding mentored tele-sessions and simulation-based practice can accelerate skill acquisition and confidence. As self-reported readiness often fails to predict actual use, services should pair training with systematic audits of objective indicators (e.g., number of remote OEP initiations, adherence rates, and delivered dosage).

PT curricula at entry-to-practice and postgraduate levels should integrate digital-health competencies across the continuum, with explicit instruction in fall epidemiology, OEP dosing and progression, remote safety checks, and video-based performance measures. Capstone projects and clinical rotations that include remote delivery can normalize hybrid models early in professional formation. These directions align with WHO strategies that emphasize workforce capacity building as foundational for scaling digital health [15].

Translating individual readiness into sustained practice requires organizational and policy infrastructure. Health system administrators should ensure secure, interoperable platforms; standardized documentation frameworks; reimbursement that recognizes remote fall-prevention services as billable encounters; and accessible technology with appropriate training for older adults and caregivers. The WHO PIR provides service-level resourcing guidance, and Rehabilitation 2030 underscores the intersectoral partnerships needed to extend access to underserved populations [29].

Within the KSA, a prior national work established strong psychometric properties for instruments measuring the OEP knowledge and attitudes among PTs and provided baseline data on professional perspectives [5]. This study identifies theory-driven predictors of belief in telerehabilitation efficacy and readiness to implement remote fall prevention, highlighting two actionable targets: specialized fall-prevention training and international educational exposure. Ministries of health and higher education, academic institutions, and professional licensing bodies can leverage these determinants to develop a digitally competent rehabilitation workforce capable of delivering safe, evidence-based telerehabilitation for fall prevention. As the KSA confronts the clinical and economic implications of rapid population aging, these findings offer direction for workforce strategies that expand equitable access to fall-prevention services through scalable digital care models.

### 4.7. Future Research

To address the implementation of remote occupational exercise programs (OEP) for older adults, it is crucial to conduct adequately powered prospective studies that follow physical therapists over time. These studies should aim to test sequence effects: targeted training increases beliefs, which in turn increase readiness, leading to the prescription of remote OEP and subsequent patient enrollment. To strengthen causal inferences, it is important to include objective implementation outcomes, such as the number of remote OEP initiations, adherence rates, and delivered dosages.

Furthermore, multilevel implementation evaluations guided by the Consolidated Framework for Implementation Research (CFIR) and the RE-AIM framework should be undertaken to identify both inner- and outer-setting determinants. Key factors to consider include leadership support, IT infrastructure, reimbursement policies, and the availability of digital-literacy resources for older adults. These evaluations will provide insights into how individual readiness translates into sustained practice change.

In addition, it is essential to assess the effectiveness and safety of tele-delivered OEP within real-world settings. This assessment should focus on patient-level metrics, including adherence to programs, falls and near-falls, balance measurements, and patient-reported outcomes. Pragmatic studies, such as StandingTall, demonstrate the potential of remote balance exercise programs, and similar trials in Saudi settings can further evaluate aspects of effectiveness, acceptability, and cost-effectiveness [36].

Lastly, future studies utilizing the UTAUT in this population should incorporate validated scales for the remaining original constructs, specifically Effort Expectancy and Social Influence. This inclusion will help quantify their contributions to intention, Performance Expectancy, and overall readiness for engaging with the remote OEP.

## 5. Conclusions

Specialized fall-prevention training and international educational exposure—proxies for robust Facilitating Conditions—emerged as the key correlates of belief in telerehabilitation efficacy and readiness to implement the OEP among Saudi PTs. At the same time, familiarity alone did not predict use. These results, consonant with the APTA guidance and WHO system-level frameworks, highlight the need to move beyond the awareness of competency-based preparation and organizational enablement to scale evidence-based fall-prevention interventions. Health system leaders, educators, and professional societies should prioritize structured upskilling, curricular reform that embeds digital-health competencies, and policies that support safe, reimbursable remote care. Larger, longitudinal, and multilevel studies are warranted to refine effect sizes, clarify mechanisms, and ensure that clinician readiness translates into sustained adoption and improved outcomes for older adults at risk of falls.

## Figures and Tables

**Table 3:** Use of the OEP by telerehabilitation familiarity.

Previously Prescribed OEP	Familiar (Yes), *n* (%)	Familiar (No), *n* (%)	Total
No	64 (76.2%)	29 (80.6%)	93
Yes	20 (23.8%)	7 (19.4%)	27
Total	84	36	120
Statistical Test	χ^2^(1) = 0.28, *p* = 0.597		

Abbreviation: OEP, Otago Exercise Program.

**Table 4:** Ordinal logistic regression for belief (*n* = 117).

Predictor	OR	95% CI (Lower)	95% CI (Upper)	*p*-Value
Familiarity (Yes vs. No)	0.921	0.410	2.068	0.841
Training (Yes vs. No)	3.997	1.568	10.184	0.004
Experience (6–10 y vs. <5 y)	1.897	0.727	4.951	0.191
Experience (>10 y vs. <5 y)	1.633	0.631	4.227	0.312

Abbreviations: OR, odds ratio; CI, confidence interval.

**Table 5:** Logistic regression for high readiness (N = 117; 21 events).

Predictor	OR	95% CI (Lower)	95% CI (Upper)	*p*-Value
Knowledge (Low vs. High)	5.829	0.620	54.833	0.123
Knowledge (Moderate vs. High)	10.309	1.276	83.336	0.029
Belief (High vs. Low)	8.680	0.884	85.229	0.064
Belief (Moderate vs. Low)	3.069	0.195	48.380	0.425
Training (Yes vs. No)	4.520	1.546	13.210	0.006
Experience (6–10 y vs. <5 y)	0.981	0.225	4.276	0.980
Experience (>10 y vs. <5 y)	0.740	0.171	3.212	0.688
University (Outside vs. Local)	5.170	1.435	18.636	0.012
Region (Central vs. Southern)	1.728	0.366	7.388	0.460
Region (Eastern vs. Southern)	4.010	0.192	83.874	0.371
Region (Western vs. Southern)	1.486	0.277	7.973	0.644
Region (Northern vs. Southern)	1.302	0.221	7.669	0.770

Abbreviations: OR, odds ratio; CI, confidence interval.

## Data Availability

Data are available upon reasonable request to the corresponding author.

## Supplementary Materials

The following supporting information can be downloaded at https://www.mdpi.com/article/10.3390/jcm14217838/s1, Table S1. Sensitivity analyses for the readiness outcome (high readiness vs. not). Table S2. Variance inflation factors (VIFs) for the extended readiness model.

## Abbreviations

The following abbreviations are used in this manuscript:

Abbreviation	Expansion
AIC	Akaike Information Criterion
APTA	American Physical Therapy Association
CFIR	Consolidated Framework for Implementation Research
CI	Confidence interval
DPT	Doctor of Physical Therapy
ICC	Intraclass correlation coefficient
IT	Information technology
KSA	Kingdom of Saudi Arabia
OEP	Otago Exercise Program
OR	Odds ratio
PIR	Package of Interventions for Rehabilitation
PhD	Doctor of Philosophy
PT	Physical therapy/physiotherapy
PTs	Physical therapists/physiotherapists
RE-AIM	Reach, Effectiveness, Adoption, Implementation, Maintenance
SPSS	Statistical Package for the Social Sciences
UTAUT	Unified Theory of Acceptance and Use of Technology
VIF	Variance inflation factor(s)
WHO	World Health Organization
