# Supplementary material for: Telerehabilitation for Fall Prevention in Saudi Arabia: Readiness and Predictors Among Physical Therapists
Source: J Clin Med. 2025 Nov 4;14(21):7838. doi: 10.3390/jcm14217838 (PMC12608178; doi:10.3390/jcm14217838)
Supplement: Supplementary file 1 [file jcm-14-07838-s001.zip › jcm-3943798-supplementary.pdf]

Supplementary Materials

S1. Sensitivity analyses for the readiness outcome

S1.1 Rationale and approach

Because the number of “high-readiness” events was small (n = 21), we stress-tested the **readiness** model (binary outcome) using two sensitivity analyses requested by Reviewer 2:

1. Firth’s penalized logistic regression to mitigate small-sample bias and potential separation; and
2. Pilot-exclusion analysis, re-estimating the model after removing pilot respondents (n = 30), leaving the main-phase sample (n = 87; readiness events = 16).

Both sensitivities used the same covariates as the main readiness model and reported odds ratios (OR) with exact *p*-values.

S1.2 Results summary

Findings were directionally consistent and remained statistically significant for the two a priori predictors of interest—fall-prevention training and education outside Saudi Arabia—across all specifications (Table S1).

Table S1. Sensitivity analyses for the readiness outcome (high readiness vs not)

Predictor	Original model (logistic) OR (*p*)	Firth’s penalized logistic OR (*p*)	No-pilot logistic OR (*p*)
Fall-prevention training (Yes vs No)	4.52 (0.006)	3.74 (0.010)	4.85 (0.025)
University outside vs local	5.17 (0.012)	4.15 (0.018)	5.35 (0.037)

Notes.

1. The main readiness model excludes *belief* to reduce circularity; knowledge categories were retained (see Methods §2.5, §2.7).
2. “Firth” = penalized likelihood logistic regression; “No-pilot” = model re-fit in main-phase respondents only (n = 87; 16 events).
3. All models adjust for the prespecified covariates used in the main analysis. Interpretation remains exploratory/hypothesis-generating given small event counts.

S1.3 Interpretation

Firth’s penalization yielded attenuated but still significant estimates for training (OR = 3.74; $p = 0.010$) and education outside Saudi Arabia (OR = 4.15; $p = 0.018$), supporting robustness to small-sample bias. Excluding pilot respondents produced consistent results (training OR = 4.85; $p = 0.025$; education OR = 5.35; $p = 0.037$) despite fewer events, indicating the main findings are not driven by the pilot subgroup.

Table S2. Variance Inflation Factors (VIFs) for the extended readiness model.

**Predictor (readiness model)**	**VIF**
Knowledge (Low vs High)	1.3414
Knowledge (Moderate vs High)	1.2721
Belief (High vs Low)	1.2588
Belief (Moderate vs Low)	1.1347
Training (Yes vs No)	1.2120
Experience (6–10 y vs <5 y)	1.2575
Experience (>10 y vs <5 y)	1.3069
University (Outside vs Local)	1.7201
Region (Central vs Southern)	1.6385
Region (Eastern vs Southern)	1.1408
Region (Western vs Southern)	1.3653
Region (Northern vs Southern)	1.3440

Note. VIF < 5 indicates acceptable collinearity; VIF < 2 is preferred for small-event models. All VIFs were < 2, suggesting low multicollinearity.
